# Motoric Cognitive Risk Syndrome Associated With Risk of Frailty and Likelihood of Reversion in Older Adults

**DOI:** 10.1002/jcsm.70033

**Published:** 2025-07-29

**Authors:** Dingchun Hou, Bo Liang, Lijun Pei, Gong Chen

**Affiliations:** ^1^ Institute of Population Research, Peking University Beijing China; ^2^ Institute of Ageing Studies, Peking University Beijing China

**Keywords:** frailty, motoric cognitive risk syndrome, reversibility

## Abstract

**Background:**

Motoric cognitive risk syndrome (MCR), a predementia condition, is reported to be associated with frailty. However, the associations of MCR with frailty risk and its reversibility remain unclear.

**Methods:**

A longitudinal study was conducted of 10 809 older adults from the Health and Retirement Study in the United States. Frailty was assessed by the frailty index and reversibility was measured by transitions from frailty at baseline to non‐frailty during follow‐up. MCR was defined as the presence of both subjective cognitive complaints and slow gait speed in individuals without dementia or mobility disability. Multistate Markov models were performed to evaluate the effects of MCR on transitions among non‐frailty, frailty and death. Cox regression models were used to estimate MCR associated with the risk of frailty and the likelihood of reversion.

**Results:**

Among 10 809 participants with a median age of 71 (interquartile range: 67–71) years and 6462 (57.93%) females, a total of 105 372 person‐years (9.75 years in average) were followed up. The prevalence of MCR at baseline in non‐frail and frail participants was 3.12% and 18.56%, respectively. Compared with non‐MCR, MCR increased the probability of transitioning from non‐frailty to frailty by 46% (HR = 1.46, 95% CI: 1.27–1.67) and decreased the probability of transitioning from frailty to non‐frailty by 36% (HR = 0.64, 95% CI: 0.52–0.77). Compared with normal function, subjective cognitive complaints only, slow gait speed only and MCR increased the probability of transitioning from non‐frailty to frailty by 43% (HR = 1.43, 95% CI: 1.33–1.53), 14% (HR = 1.14, 95% CI: 1.00–1.30) and 69% (HR = 1.69, 95% CI: 1.47–1.96) and decreased the probability of transitioning from frailty to non‐frailty by 18% (HR = 0.82, 95% CI: 0.74–0.92), 27% (HR = 0.73, 95% CI: 0.60–0.88) and 45% (HR = 0.55, 95% CI: 0.45–0.67), respectively. As shown in the prospective analyses, MCR and its single component were associated with increased frailty risk and decreased likelihood of reversion.

**Conclusions:**

MCR is associated with an increased risk of frailty, accelerated transition from non‐frailty to frailty and a decreased likelihood of reversion from frailty. MCR emerges as a promising risk stratification factor for frailty, highlighting the need for increased focus on individuals with MCR, particularly within the pre‐frail population.

**Abbreviations**
APOE: apolipoprotein E
BMI: body mass index
CI: confidence interval
CRP: C‐reactive protein
FI: frailty index
HbA1c: glycosylated haemoglobin A1c
HDL‐C: high‐density lipoprotein cholesterol
HR: hazard ratio
HRS: health and retirement study
IQR: interquartile range
MCR: motoric cognitive risk syndrome
PEF: peak expiratory flow
RCS: restricted cubic spline
SCC: subjective cognitive complaints
TC: total cholesterol
VIF: variance inflation factor

## 1 Introduction

Frailty, a dynamic degenerative aging process, is characterized by a condition of diminished physiological reserve of numerous systems and reduced physical and mental resilience to stress [1, 2]. Frailty is strongly linked to mortality and is a reflection of biological and phenotypic factors rather than chronological age [3, 4]. Frailty risk increases with age, significantly increasing the susceptibility to adverse health outcomes such as falls, disability and mortality [1, 2]. However, in certain patients, especially in the early stages, frailty may be reversible [1, 2, 3]. Therefore, clinical care, early interventions and prognosis may be affected if frailty and its modifiable associated factors are identified in older adults.

MCR is a predementia syndrome defined by subjective cognitive complaints (SCC) and slow gait speed among individuals without dementia or mobility impairments [5]. Globally, nearly 10% of older adults are affected by MCR, and in the United States, its prevalence is about 7% [6, 7, 8]. MCR is essentially characterized as a specific, cognitively‐oriented risk state indicating elevated vulnerability to dementia. It is closely associated with cerebrovascular and fronto‐subcortical dysfunction. Frailty, by comparison, manifests as a broader syndrome of multisystem physiological decline resulting in reduced resilience, identified through a constellation of physical and energetic deficits [9].

The critical nexus between MCR and frailty is evidenced by their shared component of slow gait speed, overlapping risk factors, common pathophysiological mechanisms—particularly those involving vascular and inflammatory pathways—their frequent co‐occurrence and their shared prognostic significance for adverse outcomes in older adults [9, 10, 11]. Frailty represents a more advanced, severe and globally compromised state arising from the cumulative burden of multisystem physiological deficits [12]. It occurs further downstream in the aging‐disease continuum, marking a critical transition point toward disability and dependency [9, 12]. Conversely, MCR is positioned earlier in this continuum, serving as a specific, cognitively focused warning sign. It identifies individuals poised at the threshold of significant decline, primarily signalling heightened risk for dementia but also indicating potential progression toward physical frailty [13]. This progression is facilitated by shared underlying mechanisms and the gateway deficit of slow gait speed. Furthermore, MCR has international appeal, due to the ease of implementing SCC and gait assessment, which is virtually cost free and requires limited training [5, 14]. Clinically, the recognition of MCR provides a vital window of opportunity for implementing preventive interventions aimed at arresting the progression toward the more extensive and severe health deficits characteristic of frailty. This distinction is crucial for developing targeted therapeutic approaches that address the specific pathophysiological trajectories of each condition while acknowledging their interconnected nature.

A cross‐sectional study suggested that MCR may serve as a superior predictor of frailty compared to its single component [15]. Prior research indicated that frailty was associated with an increased risk of MCR [16]. These findings may suggest potential bidirectional associations and shared underlying mechanisms between them. However, the absence of prospective studies limits the ability to determine whether MCR can increase the risk of frailty. Furthermore, although there is a growing interest in the reversibility of frailty, there remains a scarcity of research addressing the association between MCR and the reversibility of frailty. The reversibility of frailty is a critical area of research, and MCR, due to its earlier onset and ease of measurement, could offer valuable insights into the modifiable factors associated with frailty outcomes [17, 18]. It is significant to focus on elucidating the role of MCR in frailty reversibility and its potential as a therapeutic target for interventions aimed at improving health outcomes in frail populations.

In this study, we hypothesized that MCR is associated with an increased risk of frailty and a reduced likelihood of frailty reversion. To test this hypothesis, a longitudinal study was conducted to estimate the associations of MCR with the risk of frailty and the likelihood of reversion in older adults using a nationwide population‐based longitudinal survey database from the United States.

## 2 Methods

### 2.1 Study Population

A longitudinal study was conducted in our study. The data was from the health and retirement study (HRS) from the United States, and detailed information of study design for the longitudinal survey has been published previously [19]. In brief, the HRS, a nationwide, prospective, population‐based longitudinal study, was approved by the Ethics Review Committees of the University of Michigan. Informed consent was obtained from each participant in the survey.

In the HRS, Wave 8 (2006) to Wave 13 (2016) was used as the baseline, with biennial follow‐up surveys continuing until Wave 15 (2020). Participants were included if they were aged 65 years or older and had complete data on MCR at baseline. Individuals with missing data for 10% or more items of frailty index at baseline or during follow‐up were excluded. FI was assessed every 2 years during the follow‐up period. Finally, 10 809 participants from the HRS were included in the multistate transition analysis between MCR and frailty. After excluding 2651 participants who were frail at baseline, 8158 participants were included for the prospective analysis examining the association between MCR and frailty risk. The 2651 participants who were frail at baseline were included in the analysis assessing the association between MCR and frailty reversibility. The flowchart depicting the sample selection process is shown in Figure S1.

### 2.2 Assessment of Frailty

Frailty was assessed using the frailty index (FI), which is calculated based on the accumulation of age‐related health deficits [20, 21]. In this study, we constructed the FI in accordance with the standard procedures previously described [21]. After screening the data from the HRS, we selected 30 items to construct the FI, encompassing variables related to comorbidity, physical function, disability, depression and cognition (Table S1). Each item was dichotomized into 0 or 1 based on specific cut‐off values, with the exception of the cognition item. A value of 0 indicated the absence of a deficit, while 1 indicated its presence. The cognition item was treated as a continuous variable, ranging from 0 to 1, where a higher value signified poorer cognitive function. Consistent with previous studies, the FI was used to categorize participants into two groups: non‐frailty (FI < 0.25) and frailty (FI ≥ 0.25) [20]. The FI was calculated for each wave of the HRS datasets. Given the low proportion of missing data for each FI item, random forest imputation using the missRanger package in R software was employed to maximize the sample size [22]. The reversibility of frailty was assessed by tracking transitions from a state of frailty at baseline to a state of non‐frailty during the follow‐up period.

### 2.3 Access to Death Data

The date of death for participants was ascertained through interviews with family members, who were contacted at each follow‐up investigation.

### 2.4 Assessment of Motoric Cognitive Risk Syndrome

Motoric cognitive risk syndrome (MCR) was defined as the presence of both subjective cognitive complaints and slow gait speed in individuals without dementia or mobility disability [5]. Data for MCR were collected at baseline. In the HRS, two questions were used to assess subjective cognitive complaints: ‘How would you rate your memory at the present time? Would you say it is excellent, very good, good, fair, or poor’? and ‘Compared with the previous interview, would you say your memory is better now, about the same, or worse than it was then’? Subjective cognitive complaints were represented by a response of ‘fair’ or ‘poor’ on the first item or ‘worse’ on the second item. Gait speed was measured using a 2.5‐m test. Slow gait speed was defined as gait speed ≥ 1 standard deviation below age‐specific (65–74 years or ≥ 75 years) and sex‐specific (men or women) means within each wave. The specific cutoffs for slow gait speed are presented in Table S1. Dementia was identified by either of the following criteria: report by the participant or a proxy that a doctor had diagnosed the participant with dementia or Alzheimer's disease, or a score of 6 or lower on a composite score from tasks including immediate and delayed recall, serial sevens and backward counting [23]. Individuals were diagnosed with MCR when they presented both subjective cognitive complaints (in the absence of dementia) and slow gait speed [5].

### 2.5 Assessment of Covariates

The covariates included age, sex, race, place of residence, educational level, marital status, per capita household income, smoking status, alcohol consumption, physical activity, social participation, body mass index (BMI), waist circumference, pain, tooth loss, hospitalization history, medication use, handgrip strength, peak expiratory flow (PEF), systolic blood pressure, diastolic blood pressure, high‐density lipoprotein cholesterol (HDL‐C), total cholesterol (TC), C‐reactive protein (CRP), glycosylated haemoglobin A1c (HbA1c) and apolipoprotein E (APOE) gene. Measurements and cutoffs for covariates were presented in Table S1. Data for all covariates were collected at baseline. Given the low proportion of missing data for covariates, random forest imputation using the missRanger package in R software was applied [22].

### 2.6 Statistical Analysis

For descriptive analysis, continuous variables that were not normally distributed were presented as median and interquartile range (IQR). Categorical variables were presented as frequency and percentage.

In this study, we performed statistical inference through three distinct steps. First, three‐state Markov models, which are effective for describing processes where individuals transition between states over continuous time, were utilized for multistate analysis [24]. Markov models were used to estimate the associations between MCR and transitions among non‐frailty, frailty and death states. Second, to mitigate the risk of reverse causality, we established prospective cohorts and utilized Cox proportional hazards regression models to assess the relationship between baseline MCR exposure and follow‐up frailty outcome (Analysis 2). Finally, to confirm the association between MCR and frailty reversibility, we constructed prospective cohorts with non‐frailty as the outcome and applied Cox proportional hazard regression models to estimate the association between MCR and the probability of non‐frailty (Analysis 3). The proportional hazards assumptions were verified using tests based on Schoenfeld residuals, and no violations of the assumption were observed.

Furthermore, to investigate whether exposure‐outcome association varies across subgroups, thereby assessing the heterogeneity of effects, we conducted subgroup analyses. All models referenced the non‐MCR or normal function, adjusted for statistically significant baseline covariates in the univariate analysis and presented results as hazard ratios (HR) with 95% confidence intervals (95% CI). To minimize potential temporal confounding and birth cohort effects, baseline assessment time was statistically adjusted. Variance inflation factor (VIF) was calculated to test multicollinearity, and VIF for each variable was < 5. For additional robustness checks, especially for the Markov models, we transformed continuous covariates including handgrip strength and PEF into categorical variables based on their quartiles.

To ascertain the robustness of our findings, we conducted several sensitivity analyses: (1) Given that the FI cut‐off values for defining frailty status were controversial, we used another common cut‐off value to define frailty status, with frailty defined as FI > 0.21 and non‐frailty defined as FI ≤ 0.21 [20]; (2) To minimize reverse causality, the prospective analyses were repeated after excluding participants with less than 3 years of follow‐up. (3) We further divided participants into robustness (FI ≤ 0.10), pre‐frailty (0.10 < FI < 0.25), and frailty (FI ≥ 0.25) to repeat our analysis; (4) Prior research indicated that MCR and mild cognitive impairment (MCI) were both intermediate conditions between natural aging and dementia and individuals with MCR may be combined with MCI [25]. To reduce the impact of MCI on the subjective cognitive function measurement, we further excluded participants with MCI at baseline to repeat our analyses for sensitivity analyses. The criteria for MCI was shown in Table S1; (5) Given the availability of an internationally recognized threshold, 0.8 m/s was used as the cut‐off value of slow gait speed for sensitivity analyses [26].

All analyses were performed using STATA, version 17.0 and R software, version 4.4.1. *p* < 0.05 (two‐tailed) was considered statistically significant.

## 3 Results

### 3.1 Baseline and Follow‐Up Characters of Study Population

A total of 10 809 eligible participants with a median age of 71 (IQR: 67–71) years and 6462 (57.93%) females were included in this analysis. Among American older adults, the overall prevalence of MCR was 6.90%. When stratified by frailty status, baseline prevalences were 3.12% for non‐frail individuals and 18.56% for frail individuals. We observed 4551 transitions from non‐frailty to frailty and 1885 transitions from frailty to non‐frailty across 8 waves; 1077 and 2442 participants transitioned to death from non‐frailty and frailty, respectively. For Analysis 2, 8158 eligible participants were included and followed up for a total of 57 366 person‐years, with an incidence density of frailty at 61.43 per 1000 person‐years. For Analysis 3, 2651 eligible participants were included and followed up for 14 508 person‐years, with an incidence density of non‐frailty at 50.52 per 1000 person‐years. The baseline characters of the study population for the multistate transition analysis are detailed in Table 1.

**TABLE 1:** Distribution characteristics of frailty among American older adults at baseline (*n* = 10 809).

Characteristics	Non‐frailty (*n* = 8158) *n* (%)	Frailty (*n* = 2651) *n* (%)	*P*‐value
Age (years)			< 0.001
≥ 65 and < 75	5681 (69.64)	1566 (59.07)	
≥ 75	2477 (30.36)	1085 (40.93)	
Sex			< 0.001
Male	3604 (44.18)	943 (35.57)	
Female	4554 (55.82)	1708 (64.43)	
Race			< 0.001
White	6923 (84.86)	2039 (76.91)	
Black	921 (11.29)	468 (17.65)	
Others	314 (3.85)	144 (5.44)	
Place of residence			0.012
Urban	3896 (47.76)	1179 (44.47)	
Suburban	1858 (22.78)	649 (24.49)	
Exurban	2404 (29.46)	823 (31.04)	
Educational level			< 0.001
Below high school	1497 (18.35)	1006 (37.95)	
High school and above	6661 (81.65)	1645 (62.05)	
Marital status			< 0.001
Married or partnered	5630 (69.01)	1495 (56.39)	
Others	2528 (30.99)	1156 (43.61)	
Per capita household income ($)			< 0.001
Q_1_	1658 (20.32)	1042 (39.31)	
Q_2_	1953 (23.94)	751 (28.33)	
Q_3_	2178 (26.70)	524 (19.76)	
Q_4_	2369 (29.04)	334 (12.60)	
Smoking status			< 0.001
Never smokers	3679 (45.10)	1042 (39.31)	
Former smokers	3726 (45.67)	1313 (49.53)	
Current smokers	753 (9.23)	296 (11.16)	
Alcohol consumption			< 0.001
No	3494 (42.83)	1664 (62.77)	
Yes	4664 (57.17)	987 (37.23)	
Physical activity			< 0.001
Normal	7258 (88.97)	1808 (68.20)	
Inactivity	900 (11.03)	843 (31.80)	
Social participation			< 0.001
Q_1_	1799 (22.05)	758 (28.59)	
Q_2_	1801 (22.08)	700 (26.41)	
Q_3_	2309 (28.30)	676 (25.50)	
Q_4_	2249 (27.57)	517 (19.50)	
Body mass index (kg/m^2^)			< 0.001
< 25	1960 (24.03)	420 (15.84)	
≥ 25 and < 30	3185 (39.04)	824 (31.08)	
≥ 30	3013 (36.93)	1407 (53.08)	
Waist circumference (cm)			< 0.001
Normal	2954 (36.21)	536 (20.22)	
Central obesity	5204 (63.79)	2115 (79.78)	
Pain			< 0.001
No	6201 (76.01)	975 (36.78)	
Yes	1957 (23.99)	1676 (63.22)	
Tooth loss			< 0.001
No	7514 (92.11)	2213 (83.48)	
Yes	644 (7.89)	438 (16.52)	
Hospitalization history (times)			< 0.001
0	6372 (78.11)	1533 (57.83)	
1	1288 (15.79)	606 (22.86)	
≥ 2	498 (6.10)	512 (19.31)	
Medication use (kinds)			< 0.001
0	2991 (36.66)	282 (10.64)	
1 or 2	4786 (58.67)	1620 (61.11)	
≥ 3	381 (4.67)	749 (28.25)	
Handgrip strength (kg)			< 0.001
Q_1_	1715 (21.02)	987 (37.23)	
Q_2_	2017 (24.72)	685 (25.84)	
Q_3_	2082 (25.52)	574 (21.65)	
Q_4_	2344 (28.74)	405 (15.28)	
Peak expiratory flow (L/min)			< 0.001
Q_1_	1571 (19.26)	941 (35.50)	
Q_2_	1961 (24.04)	737 (27.80)	
Q_3_	2157 (26.44)	554 (20.90)	
Q_4_	2469 (30.26)	419 (15.80)	
Systolic blood pressure (mmHg)			0.006
< 140	5527 (67.75)	1719 (64.84)	
≥ 140	2631 (32.25)	932 (35.16)	
Diastolic blood pressure (mmHg)			0.284
< 90	7004 (85.85)	2298 (86.68)	
≥ 90	1154 (14.15)	353 (13.32)	
High‐density lipoprotein cholesterol (mg/dL)			< 0.001
Normal	6281 (76.99)	1783 (67.26)	
Low	1877 (23.01)	868 (32.74)	
Total cholesterol (mg/dL)			< 0.001
< 240	7026 (86.12)	2385 (89.97)	
≥ 240	1132 (13.88)	266 (10.03)	
C‐reactive protein (mg/L)			< 0.001
≤ 3	4902 (60.09)	1295 (48.85)	
> 3	3256 (39.91)	1356 (51.15)	
Glycosylated haemoglobin A1c (%)			< 0.001
< 6.5	7229 (88.61)	1938 (73.10)	
≥ 6.5	929 (11.39)	713 (26.90)	
Apolipoprotein E gene			0.570
Non‐ε4 carriers	5807 (71.18)	1859 (70.12)	
Heterozygous ɛ4 carriers	2216 (27.16)	745 (28.10)	
Homozygous ɛ4 carriers	135 (1.65)	47 (1.78)	
Motoric cognitive risk syndrome			< 0.001
Normal	4907 (60.15)	736 (27.76)	
Subjective cognitive complaints only	2553 (31.29)	1132 (42.70)	
Slow gait speed only	444 (5.44)	291 (10.98)	
Motoric cognitive risk syndrome	254 (3.12)	492 (18.56)	

### 3.2 Associations of MCR With the Risk and Reversibility of Frailty

Associations between MCR and transitions among non‐frailty, frailty and death were estimated using Markov models (Table 2). Compared with non‐MCR, MCR increased the probability of transitioning from non‐frailty to frailty by 46% (HR = 1.46, 95% CI: 1.27–1.67), and decreased the probability of transitioning from frailty to non‐frailty by 36% (HR = 0.64, 95% CI: 0.52–0.77). However, the associations between MCR and transitioning from non‐frailty or frailty to death were not statistically significant (*p* > 0.05). Compared with normal function, SCC only, slow gait speed only and MCR increased the probability of transitioning from non‐frailty to frailty by 43% (HR = 1.43, 95% CI: 1.33–1.53), 14% (HR = 1.14, 95% CI: 1.00–1.30) and 69% (HR = 1.69, 95% CI: 1.47–1.96) and decreased the probability of transitioning from frailty to non‐frailty by 18% (HR = 0.82, 95% CI: 0.74–0.92), 27% (HR = 0.73, 95% CI: 0.60–0.88) and 45% (HR = 0.55, 95% CI: 0.45–0.67), respectively. SCC only decreased the probability of transitioning from frailty to death by 14% (HR = 0.86, 95% CI: 0.78–0.95). While SCC only was not associated with the probability of transitioning from non‐frailty to death (*p* > 0.05). Neither slow gait speed only nor MCR exhibited statistically significant associations with these mortality transitions (*p* > 0.05).

**TABLE 2:** Hazard ratio of motoric cognitive risk syndrome with transitions among non‐frailty, frailty, and death in the three‐state Markov models, HR (95% CI) (*n* = 10 809).

Transitions	Motoric cognitive risk syndrome					
	Non‐MCR	MCR	Normal	SCC only	Slow gait speed only	MCR
State 1 to State 2	Ref.	**1.46 (1.27–1.67)**	Ref.	**1.43 (1.33–1.53)**	**1.14 (1.00–1.30)**	**1.69 (1.47–1.96)**
State 1 to State 3	Ref.	0.68 (0.17–2.77)	Ref.	0.69 (0.34–1.38)	1.11 (0.56–2.18)	0.54 (0.09–3.07)
State 2 to State 1	Ref.	**0.64 (0.52–0.77)**	Ref.	**0.82 (0.74–0.92)**	**0.73 (0.60–0.88)**	**0.55 (0.45–0.67)**
State 2 to State 3	Ref.	0.94 (0.83–1.07)	Ref.	**0.86 (0.78–0.95)**	1.04 (0.90–1.20)	0.89 (0.77–1.03)

*Note:* Boldface: *p* < 0.05. State 1: non‐frailty; State 2: frailty; State 3: death. HR: hazard ratio after adjusting for age, sex, race, place of residence, educational level, marital status, per capita household income, smoking status, alcohol consumption, physical activity, social participation, body mass index, waist circumference, pain, tooth loss, hospitalization history, medication use, hand grip strength, peak expiratory flow, systolic blood pressure, diastolic blood pressure, high‐density lipoprotein cholesterol, total cholesterol, C‐reactive protein, glycosylated haemoglobin A1c, apolipoprotein E gene and baseline assessment time.

Abbreviations: CI: confidence interval; MCR: motoric cognitive risk syndrome; SCC: subjective cognitive complaints.

To further examine the prospective associations of MCR with the risk and reversibility of frailty, we constructed prospective cohorts with frailty and non‐frailty as outcomes. The Cox proportional hazard regression model was employed for these analyses (Table 3). Compared with non‐MCR, MCR increased the risk of frailty by 39% (HR = 1.39, 95% CI: 1.18–1.65) and decreased the probability of non‐frailty by 47% (HR = 0.53, 95% CI: 0.42–0.68). Compared with normal function, SCC only, slow gait speed only and MCR increased the risk of frailty by 47% (HR = 1.47, 95% CI: 1.37–1.58), 19% (HR = 1.19, 95% CI: 1.03–1.37) and 63% (HR = 1.63, 95% CI: 1.38–1.93) and decreased the probability of non‐frailty by 21% (HR = 0.79, 95% CI: 0.67–0.94), 27% (HR = 0.73, 95% CI: 0.56–0.96) and 55% (HR = 0.45, 95% CI: 0.34–0.59), respectively.

**TABLE 3:** Associations of motoric cognitive risk syndrome with frailty risk and its reversibility in the Cox proportional hazard regression models, HR (95% CI).

Motoric cognitive risk syndrome	Frailty (*n* = 8158)	Frailty reversibility (*n* = 2651)
**Non‐MCR**	Ref.	Ref.
MCR	**1.39 (1.18–1.65)**	**0.53 (0.42–0.68)**
Normal	Ref.	Ref.
Subjective cognitive complaints only	**1.47 (1.37–1.58)**	**0.79 (0.67–0.94)**
Slow gait speed only	**1.19 (1.03–1.37)**	**0.73 (0.56–0.96)**
MCR	**1.63 (1.38–1.93)**	**0.45 (0.34–0.59)**

*Note:* Boldface: *p* < 0.05. With frailty as the outcome, hazard ratios were adjusted for age, sex, race, place of residence, educational level, marital status, per capita household income, smoking status, alcohol consumption, physical activity, social participation, body mass index, waist circumference, pain, tooth loss, hospitalization history, medication use, hand grip strength, peak expiratory flow, systolic blood pressure, high‐density lipoprotein cholesterol, total cholesterol, C‐reactive protein, glycosylated haemoglobin A1c and baseline assessment time. With non‐frailty as the outcome, hazard ratios were adjusted for age, sex, race, place of residence, educational level, marital status, per capita household income, alcohol consumption, physical activity, social participation, body mass index, pain, tooth loss, hospitalization history, medication use, hand grip strength, peak expiratory flow, total cholesterol and glycosylated haemoglobin A1c.

Abbreviations: HR: hazard ratio; CI: confidence interval; MCR: motoric cognitive risk syndrome.

**FIGURE 1:**
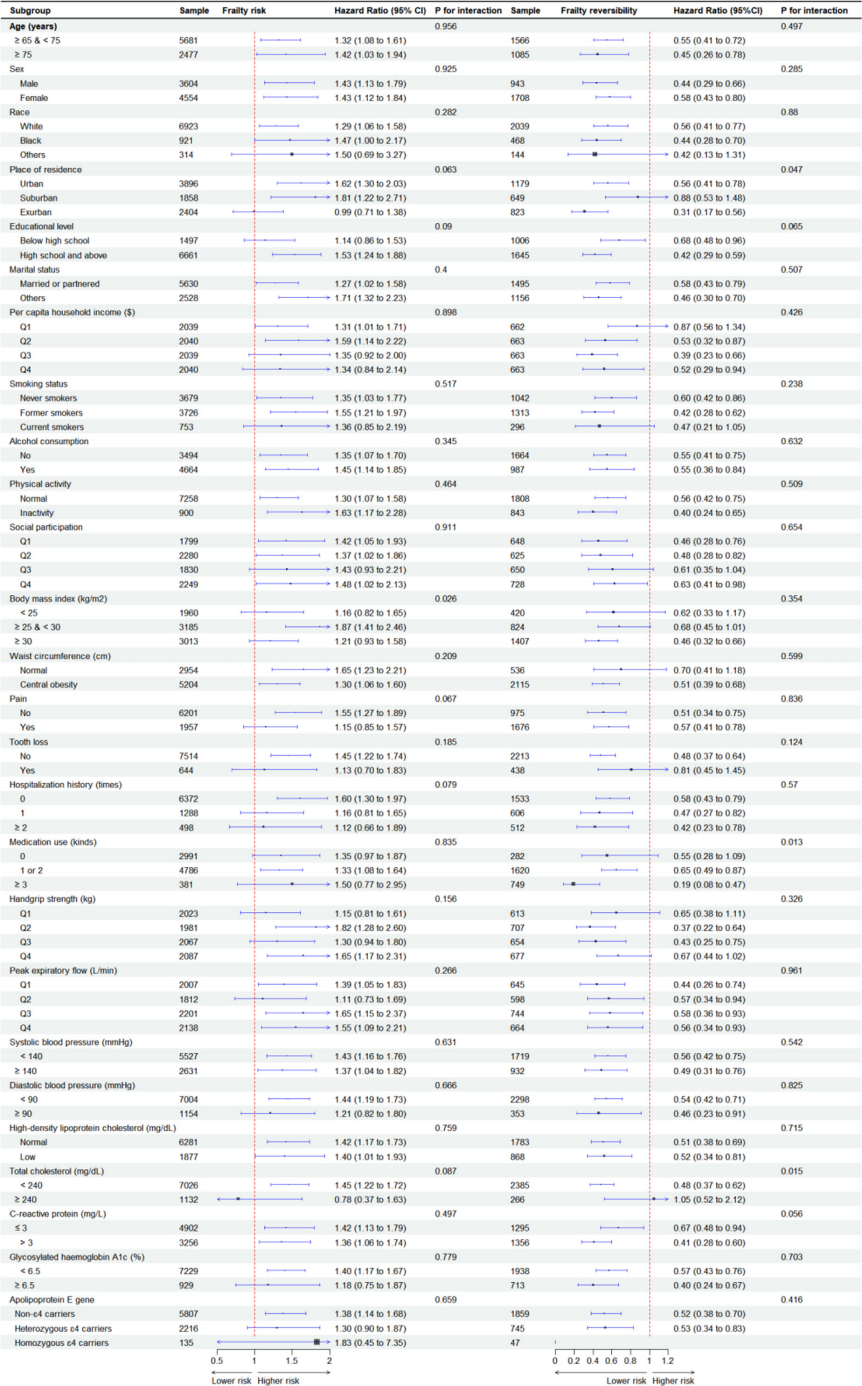
Subgroup analysis of the association of motoric cognitive risk syndrome with frailty risk and its reversibility in American older adults.

The findings from subgroup analyses were mostly consistent with the main analysis (Figure 1). BMI (*p* = 0.026) interacted with MCR on the risk of frailty. Place of residence (*p* = 0.047), medication use (*p* = 0.013) and TC levels (*p* = 0.015) interacted with MCR on the frailty reversibility.

### 3.3 Sensitivity Analyses

When we used another common cut‐off value to define frailty status, the results of multistate Markov models and prospective analyses were largely in line with the main analyses (Tables S2 and S3). However, even though the association between slow gait speed only and the probability of transitioning from non‐frailty to frailty was not statistically significant (*p* > 0.05), the prospective association between slow gait speed only and the risk of frailty was still statistically significant (HR = 1.16, 95% CI: 1.01–1.35). When we excluded participants with less than 3 years of follow‐up to conduct prospective analyses, the associations of MCR with the risk and reversibility of frailty were consistent with the main analysis (Table S4). While SCC only was not associated with the reversibility of frailty, slow gait speed only was not associated with the risk and reversibility of frailty (*p* > 0.05). When participants were categorized into robustness, pre‐frailty and frailty groups, multistate transition analysis revealed that MCR was associated with a decreased likelihood of transitioning from pre‐frailty to robustness and from frailty to pre‐frailty, while simultaneously increasing the probability of transitioning from pre‐frailty to frailty. However, the association between MCR and the probability of transitioning from robustness to pre‐frailty was not statistically significant (Table S5). Cox proportional hazard regression models further corroborated these findings and demonstrated that MCR was associated with an increased risk of frailty among initially robust participants (Table S6). When excluding participants with MCI at baseline, the results of multistate Markov models and prospective analyses were almost in line with the main analyses (Tables S7 and S8). While slow gait speed only was not associated with the reversibility of frailty (*p* > 0.05). When using 0.8 m/s as the cut‐off value of slow gait speed, the results of multistate Markov models and prospective analyses were almost in line with the main analyses (Tables S9 and S10). However, the prevalence of MCR was 20.01%, and slow gait speed only was not associated with the reversibility of frailty (*p* > 0.05). Overall, sensitivity analyses confirmed the robustness of the main analysis results.

## 4 Discussion

To the best of our knowledge, this is a large‐scale longitudinal study for the association between MCR and the risk of frailty, the likelihood of reversion, as well as transitions among non‐frailty, frailty and death. Our findings showed that subjective cognitive complaints, slow gait speed and MCR at baseline were associated with an increased risk of frailty and a reduced likelihood of reversion from frailty to non‐frailty in older adults. Furthermore, the integration of subjective cognitive complaints and slow gait speed may exhibit superior predictive capacity compared to individual components alone in elucidating their underlying mechanisms related to frailty risk and reversibility.

Several studies have investigated the association between MCR and frailty [15, 16]. Subjective cognitive decline, which can emerge in midlife, has gained recognition as an early indicator of subsequent objective cognitive decline and dementia [27]. Slow gait speed serves as one of the earliest detectable indicators of the aging process, with links to midlife aging, geriatric functional status and lifelong brain health [17, 28]. Therefore, compared with frailty characterized by multisystem dysregulation, MCR may occupy an earlier and more upstream position in the aging process, making it a crucial target for the early prevention of dementia and functional decline [9]. A cross‐sectional study showed that MCR was associated with frailty and may be a superior predictor of frailty compared to its single component [15]. Although the evidence for the association in this study is limited, it nevertheless offers preliminary insights to test our hypothesis in prospective cohort studies. Our study further supported that MCR increased the risk of frailty. While evidence suggests potential bidirectional associations between MCR and frailty, the critical role of MCR in frailty progression warrants recognition [15, 16]. Furthermore, our study indicated that among robust older adults, MCR was not associated with the risk of pre‐frailty, but was associated with an increased risk of frailty. Unlike MCR and pre‐frailty, frailty represents a more advanced, severe and global loss of physiological and psychological resilience arising from the cumulative burden of multisystem health deficits [12]. Frailty occurs further downstream in the aging‐disease continuum and exhibits a nonlinear relationship with the increasing number of abnormal systems [9, 29].

Previous studies have documented the reversibility of frailty [30, 31]. Consistent with prior research, our study also identified reversions from frailty to non‐frailty [32, 33]. These reversions primarily involved transitions from frailty to pre‐frailty or from pre‐frailty to robustness. Our findings revealed that MCR was associated with reduced reversibility of frailty, indicating a link to diminished capacity for transitioning from pre‐frailty to robustness and from frailty to pre‐frailty. Our subgroup analyses further indicated that BMI significantly interacted with MCR regarding the risk of frailty, and TC interacted with MCR regarding frailty reversibility. Therefore, MCR not only shares underlying mechanisms with frailty progression but may also influence the reversibility of frailty through metabolic and inflammatory pathways. Given its ease of measurement, MCR may represent a simple, accessible and cost‐effective tool for routine clinical frailty management. It is particularly suited for early screening, prevention and intervention in resource‐limited settings and among pre‐frail individuals, offering a foundation for enhancing global health outcomes.

The underlying mechanisms by which MCR is linked to the risk and reversibility of frailty remain poorly understood, which may involve multiple biological pathways [16]. Inflammation may play a pivotal role in the association between MCR and frailty. Inflammatory markers have been linked to subjective cognitive complaints, slow gait speed and MCR [34, 35, 36]. Meanwhile, inflammation is a key factor in frailty pathogenesis [9, 37]. Our findings revealed a significant interaction between CRP and MCR in frailty reversion, suggesting that inflammation may serve as a shared mechanism underlying both conditions. Additionally, both MCR and frailty have been shown to elevate the risk of subsequent cognitive decline and dementia, suggesting the presence of shared neurobiological mechanisms [6, 13, 38, 39]. MCR was significantly associated with smaller volumes of the total brain tissue, thalamus, hippocampus, cerebellum, insula, supplementary motor area and inferior frontal gyrus, higher volumes of white matter hyperintensities and an increased likelihood of lacunes [11]. Reported magnetic resonance imaging correlates of frailty are characterized by reduced global or regional brain volume, an increased number of cerebral microbleeds, as well as a higher number of white matter hyperintensities [9]. In addition to the central nervous system, MCR and frailty have been linked to neuromuscular aging processes [9, 11]. Future research ought to prioritize investigating the underlying mechanisms between MCR and frailty, with particular emphasis on neuroinflammation and metabolic disorders. Furthermore, APOE ɛ4 allele is associated with an increased risk of frailty [40]. While no interaction between the APOE gene and MCR was detected in relation to frailty, our subgroup analyses revealed a robust association between MCR and both frailty risk and reversibility. These findings highlight the need for additional investigation into the complex nature of gene‐phenotype interactions in this context.

Our study had several strengths. First, the high consistency in findings between the multistate transition analysis and the prospective analysis enhanced the reliability of our results. Second, we constructed prospective cohorts with a long follow‐up period and large sample size to minimize the risk of reverse causality and bolster the generalizability of our findings. Third, our study further analyzed the association of MCR with the risk of frailty and the likelihood of reversion. This provided a significant reference for understanding underlying shared mechanisms between them and developing frailty management strategies. Our study also had several limitations that warrant discussion. First, the current operationalization of SCC within the MCR framework lacks empirical validation, despite the widespread use of self‐reported SCC measurement in prior research [7, 8]. A multi‐item, multidomain‐validated SCC questionnaire is required in future research [14]. Additionally, our study relied on a single baseline assessment of MCR, which might not adequately capture the development of MCR and its effect on frailty progression. This highlights the need for future studies to observe the developmental trajectory of MCR over longer periods. Second, there may be selection bias due to the loss of follow‐up participants resulting from withdrawal during the follow‐up process. Finally, although we adjusted for a comprehensive set of covariates, including sociodemographic factors, health behaviour factors, health status indicators, biomarkers and genetic information, unmeasured confounding factors remained unaccounted for in our analyses, which may have introduced residual confounding.

In summary, this study carries significant clinical and public health implications. First, our findings affirm that MCR may serve as a critical risk stratification factor for frailty. The easy measurement of MCR provides potential for a wide application in clinical and epidemiological settings and has the ability to overcome many barriers to assessing frailty risk in rural, under‐resourced and low‐income regions. Individuals with MCR should be prioritized for frailty prevention efforts. Pre‐frailty, a key stage of the frailty progression with greater reversibility, should be recognized. Those who are robust or pre‐frail may benefit from MCR evaluation to identify at‐risk individuals early, enabling the implementation of timely prevention measures to delay the frailty progression. Additionally, evaluating MCR in the frail population may facilitate the timely determination of frailty prognosis and offer a basis for the development and refinement of intervention programs. Our findings provided valuable insights for implementing cognitive‐exercise dual‐task training and multi‐dimensional interventions in frailty management. Future research could further focus on the effects of multi‐dimensional interventions on MCR, especially potential genetic targets and modifiable lifestyle factors related to lipid metabolism and inflammation, which may yield cost‐effective strategies for frailty prevention at preclinical stages.

## 5 Conclusions

In conclusion, MCR is associated with the risk of frailty, frailty progression, and the potential for reversibility in older adults. MCR is linked to an increased risk of frailty, accelerated transition from non‐frailty to frailty, and a decreased likelihood of reversion from frailty. Consequently, MCR emerges as a promising risk stratification factor for frailty, highlighting the need for increased focus on individuals with MCR, particularly within the pre‐frail population.

## Ethics Statement

All human and animal studies have been approved by the appropriate ethics committee and have therefore been performed in accordance with the ethical standards laid down in the 1964 Declaration of Helsinki and its later amendments.

## Consent

Informed consent for publication was obtained from all participants.

## Conflicts of Interest

The authors declare no conflicts of interest.

## Supporting information


**Table S1** Measurements and cutoffs for frailty index items, motoric cognitive risk syndrome and covariates.
**Table S2** Motoric cognitive risk frailty syndrome with transitions among non‐frailty, frailty and death in the multi‐state Markov model, HR (95% CI) (*n* = 10 809).
**Table S3** Associations of motoric cognitive risk syndrome with frailty risk and its reversibility in the Cox proportional hazard regression models, HR (95% CI).
**Table S4** Associations of motoric cognitive risk syndrome with frailty risk and its reversibility in the Cox proportional hazard regression models, HR (95% CI).
**Table S5** Motoric cognitive risk frailty syndrome with transitions among robustness, pre‐frailty, frailty and death in the multi‐state Markov model, HR (95% CI) (*n* = 10 809).
**Table S6** Associations of motoric cognitive risk syndrome with transitions among robustness, pre‐frailty and frailty in the Cox proportional hazard regression models, HR.
**Table S7** Motoric cognitive risk frailty syndrome with transitions among non‐frailty, frailty and death in the multi‐state Markov model, HR (95% CI) (*n* = 9160).
**Table S8** Associations of motoric cognitive risk syndrome with frailty risk and its reversibility in the Cox proportional hazard regression models, HR (95% CI).
**Table S9** Motoric cognitive risk frailty syndrome with transitions among non‐frailty, frailty and death in the multi‐state Markov model, HR (95% CI) (*n* = 10 809).
**Table S10** Associations of motoric cognitive risk syndrome with frailty risk and its reversibility in the Cox proportional hazard regression models, HR (95% CI).

**Figure S1** Selection flow of the study population in the HRS.

## Data Availability

Data are available to researchers on request for purposes of reproducing the results or replicating the procedure by directly contacting the corresponding author.
