# Supplementary material for: Motoric Cognitive Risk Syndrome Associated With Risk of Frailty and Likelihood of Reversion in Older Adults
Source: J Cachexia Sarcopenia Muscle. 2025 Jul 29;16(4):e70033. doi: 10.1002/jcsm.70033 (PMC12304730; doi:10.1002/jcsm.70033)
Supplement: Supplementary file 1 — Table S1 Measurements and cutoffs for frailty index items, motoric cognitive risk syndrome and covariates. Table S2 Motoric cognitive risk frailty syndrome with transitions among non‐frailty, frailty and death in the multi‐state Markov model, HR (95% CI) (n = 10 809). Table S3 Associations of motoric cognitive risk syndrome with frailty risk and its reversibility in the Cox proportional hazard regression models, HR (95% CI). Table S4 Associations of motoric cognitive risk syndrome with frailty risk and its reversibility in the Cox proportional hazard regression models, HR (95% CI). Table S5 Motoric cognitive risk frailty syndrome with transitions among robustness, pre‐frailty, frailty and death in the multi‐state Markov model, HR (95% CI) (n = 10 809). Table S6 Associations of motoric cognitive risk syndrome with transitions among robustness, pre‐frailty and frailty in the Cox proportional hazard regression models, HR. Table S7 Motoric cognitive risk frailty syndrome with transitions among non‐frailty, frailty and death in the multi‐state Markov model, HR (95% CI) (n = 9160). Table S8 Associations of motoric cognitive risk syndrome with frailty risk and its reversibility in the Cox proportional hazard regression models, HR (95% CI). Table S9 Motoric cognitive risk frailty syndrome with transitions among non‐frailty, frailty and death in the multi‐state Markov model, HR (95% CI) (n = 10 809). Table S10 Associations of motoric cognitive risk syndrome with frailty risk and its reversibility in the Cox proportional hazard regression models, HR (95% CI). [file JCSM-16-e70033-s001.docx]

**Table S1** Measurements and cutoffs for frailty index items, motoric cognitive risk syndrome, and covariates

| **Variables** | **Items** | **Definition** |
| --- | --- | --- |
| Frailty index | Description of the item |  |
|  | Self-reported physician diagnosed hypertension | Yes = 1, No = 0 |
|  | Self-reported physician diagnosed diabetes | Yes = 1, No = 0 |
|  | Self-reported physician diagnosed heart disease | Yes = 1, No = 0 |
|  | Self-reported physician diagnosed stroke | Yes = 1, No = 0 |
|  | Self-reported physician diagnosed cancer | Yes = 1, No = 0 |
|  | Self-reported physician diagnosed arthritis | Yes = 1, No = 0 |
|  | Self-reported physician diagnosed chronic lung disease | Yes = 1, No = 0 |
|  | Self-reported physician diagnosed any emotional, nervous, or psychiatric problems | Yes = 1, No = 0 |
|  | Self-reported physician diagnosed memory-related disease | Yes = 1, No = 0 |
|  | Self-reported eyesight | Poor or fair = 1, excellent, very good, or good = 0 |
|  | Self-reported hearing (while using hearing aid if appropriate) | Poor or fair = 1, excellent, very good, or good = 0 |
|  | Self-reported general health status | Poor or fair = 1,  Excellent, very good, or good = 0 |
|  | Difficulty with dressing | Yes = 1, No = 0 |
|  | Difficulty with bathing or showering | Yes = 1, No = 0 |
|  | Difficulty with eating | Yes = 1, No = 0 |
|  | Difficulty with getting in and out of bed | Yes = 1, No = 0 |
|  | Difficulty with using the toilet | Yes = 1, No = 0 |
|  | Difficulty with managing money | Yes = 1, No = 0 |
|  | Difficulty with taking medication | Yes = 1, No = 0 |
|  | Difficulty with shopping for groceries | Yes = 1, No = 0 |
|  | Difficulty with preparing meals | Yes = 1, No = 0 |
|  | Mobility: difficulty with walking 100 yards or one block | Yes = 1, No = 0 |
|  | Mobility: difficulty with getting up from a chair after sitting for long periods | Yes = 1, No = 0 |
|  | Mobility: difficulty with climbing several flights of stairs without resting | Yes = 1, No = 0 |
|  | Mobility: difficulty with lifting or carrying weights over 10 pounds | Yes = 1, No = 0 |
|  | Mobility: difficulty with picking up a coin from the table | Yes = 1, No = 0 |
|  | Mobility: difficulty with stooping, kneeling, or crouching | Yes = 1, No = 0 |
|  | Mobility: difficulty with reaching arms above shoulder level | Yes = 1, No = 0 |
|  | Depression: CESD-8 questionnaire | CESD-8 ≥ 4 = 1, < 4 = 0 |
|  | Cognition: (orientation test score + memory test score + calculation score) / 19 | Continuous variable, ranging  from 0 to 1 |
| Subjective cognitive complaints | Questions |  |
|  | How would you rate your memory at the present time? Would you say it is excellent, very good, good, fair, or poor? | Fair, or poor = 1, Excellent, very good, or good = 0 |
|  | Compared with the previous interview, would you say your memory is better now, about the same, or worse than it was then? | Worse = 1, Better, or about the same = 0 |
| Slow gait speed | Gender and Age groups |  |
|  | Men < 75 years | Gait speed < 0.67 m/s in Wave 8, < 0.65 m/s in Wave 9, < 0.66 m/s in Wave 10, < 0.68 m/s in Wave 11, < 0.68 m/s in Wave 12, < 0.64 m/s in Wave 13 |
|  | Men ≥ 75 years | Gait speed < 0.52 m/s in Wave 8, < 0.52 m/s in Wave 9, < 0.55 m/s in Wave 10, < 0.56 m/s in Wave 11, < 0.51 m/s in Wave 12, < 0.52 m/s in Wave 13 |
|  | Women < 75 years | Gait speed < 0.58 m/s in Wave 8, < 0.57 m/s in Wave 9, < 0.60 m/s in Wave 10, < 0.61 m/s in Wave 11, < 0.61 m/s in Wave 12, < 0.58 m/s in Wave 13 |
|  | Women ≥ 75 years | Gait speed < 0.45 m/s in Wave 8, < 0.43 m/s in Wave 9, < 0.46 m/s in Wave 10, < 0.47 m/s in Wave 11, < 0.47 m/s in Wave 12, < 0.45 m/s in Wave 13 |
| Cognitive function | Cognitive function was assessed with a composite score from tasks including immediate and delayed recall, serial sevens, and backward counting[1]. The total score ranges from 0 to 27, classifying individuals into dementia (0-6), cognitive impairment without dementia (7-11), or normal cognition (12-27)[1]. |  |
| Age |  | ≥ 65 and < 75 = 0, ≥ 75 = 1 |
| Sex |  | Male = 0, Female = 1 |
| Race |  | White = 0, Black = 1, Others = 2 |
| Place of residence | The place of residence was measured by the Beale codes provided in HRS’s Cross-Wave Census Region/Division and Mobility File, which are consolidated from the USDA’s Rural-Urban Continuum Codes. USDA’s full code list is available on their website. The crosswalk for HRS’s classifications is in the codebook of HRS’s Cross-Wave Census Region/Division and Mobility File. There are currently three sets of Beale codes provided: 1993, 2003, and 2013. For each wave of data, the Beale code with the closest year is used. If that value is missing, the next closest Beale code year is used. If that code is also missing, the furthest Beale code year is used. If the Beale code years are equidistant from the interview wave year, the most recent Beale code year is used first. | Urban = 0, Suburban = 1, Exurban (which also includes rural counties) = 2 |
| Marital status |  | Married, or partnered = 0, Separated, divorced, widowed, or never married = 1 |
| Per capita household income | Income measures are reported in nominal dollars. The HRS and AHEAD income components are summed to create the income measures found here in the RAND HRS Longitudinal File. For each derived income measure, a flag indicates whether any or all of its components were imputed. Individual income components and imputation flags are available in the RAND HRS Detailed Imputations File. Per capita household income refers to the total household income for the last calendar year divided by the total number of the household members. Participants were categorized into four groups based on the quartiles of per capita household income at baseline. | Q_1_ = 0, Q_2_ = 1, Q_3_ = 2, Q_4_ = 3 |
| Educational level |  | Below high school = 0, High school and above = 1 |
| Smoking status |  | Never smokers = 0, Former smokers = 1, Current smokers = 2 |
| Alcohol consumption |  | No = 0, Yes = 1 |
| Physical activity | Physical activity levels were quantified based on the frequency of engagement in vigorous, moderate, and light physical activities, which were assigned metabolic equivalent of task values of 8, 4, and 2, respectively. We derived a weighted-sum score for each participant, which represents the total energy expenditure associated with physical activities, taking into account both intensity and frequency. The weightings were determined by the frequency of physical activity engagement; daily, more than once a week, once a week, 1-3 times a month, and hardly ever or never were assigned scores of 7, 4, 1, 0.5, and 0, respectively[2]. | Normal = 0, Inactivity = 1  Inactivity is defined as total caloric expenditure in the lowest sex-specific 20% |
| Social participation | We used social contact, which is recognized as informal social participation, as an alternative measurement for social participation. Social contact was conceptualized as contact frequency with people in their social networks. A set of three items were presented three times to assess the participants’ frequency of contact (including meetings, telephone conversations, or correspondence by mail or email) with children, other family, and friends. Social contact scores with children, other family, and friends were first obtained by averaging across three methods of social interactions. Then, the overall social contact score was calculated by taking the average of the three social category-specific scores. The possible scale score and the actual score range for the sample were from 1 to 6. Higher scores indicated more frequent social contact and higher level of social participation[3]. | Q_1_ = 0, Q_2_ = 1, Q_3_ = 2, Q_4_ = 3 |
| Body mass index |  | < 25 = 0, ≥ 25 and < 30 = 1, ≥ 30 = 2 |
| Waist circumference |  | Normal: < 102 cm for men and < 88 cm for women, Central obesity: ≥ 102 cm for men and ≥ 88 cm for women |
| Pain | Are you often troubled with pain? | No = 0, Yes = 1 |
| Tooth loss | Have you lost all of your upper and lower natural permanent teeth? | No = 0, Yes = 1 |
| Hospitalization history | Participants reported the number of overnight hospital stays during the last two years. | 0 times = 0, 1 time = 1, ≥ 2 times = 2 |
| Medication use | In order to lower your blood pressure, are you now taking any medication?  In order to treat or control your diabetes/blood sugar, are you now taking medication that you swallow?  Are you now using insulin shots or a pump?  Are you now taking medication or other treatment for your lung condition?  Are you now taking or carrying medication for your heart problem?  Are you now taking or carrying medication because of your heart attack?  Are you now taking or carrying medications because of angina or chest pain?  Are you taking or carrying any medication for congestive heart failure?  Are you now taking any medications because of your stroke or its complications? | 0 kinds = 0, 1 or 2 kinds = 1, ≥ 3 kinds = 2 |
| Handgrip strength | Grip strength (kg) was measured by a spring-type hand dynamometer. Participants were asked to squeeze the meter at their maximum capacity in a standing position twice for each hand. The average of readings for dominant hand was calculated. Participants were categorized into four groups based on the quartiles of handgrip strength at baseline. | Q_1_ = 0, Q_2_ = 1, Q_3_ = 2, Q_4_ = 3 ≥ 3 = 2 |
| Peak expiratory flow | Peak expiratory flow (PEF) was assessed utilizing a Mini-Wright peak flow meter (Clement Clarke International Ltd., Harlow, U.K.) coupled with a disposable mouthpiece, under the guidance of a trained interviewer. Three measurements were conducted with a 30-second interval between each, and the maximum value was adopted for the respondent’s PEF in our analyses. Participants were categorized into four groups based on the quartiles of measured PEF at baseline. | Q_1_ = 0, Q_2_ = 1, Q_3_ = 2, Q_4_ = 3 |
| Systolic blood pressure | Trained interviewers used Omron HEM-780 Intellisense automated sphygmomanometers with ComFit cuffs to take systolic and diastolic readings from participants seated with both feet on the floor. Three sets of readings, taken between 45 and 60 seconds apart from a participant’s supported left arm with the palm facing upward, were averaged for the current analyses. Measured hypertension was defined based on mean sphygmomanometer readings and classified according to The Seventh Report of the Joint National Committee on Prevention, Detection, Evaluation, and Treatment of High Blood Pressure (JNC 7) criteria, the guideline during the period of the study: at least 140 mmHg for systolic blood pressure and/or at least 90 mmHg for diastolic blood pressure. | < 140 mmHg = 0, ≥ 140 mmHg = 1 |
| Diastolic blood pressure |  | < 90 mmHg = 0, ≥ 90 mmHg = 1 |
| C-reactive protein (CRP) | Blood chemistries were collected by HRS interviewers using a series of dried blood spots (DBS) that were placed on cards and shipped to either the University of Vermont or the University of Washington to be assayed. CRP cutpoints follow a joint report released by the American Heart Association and Centers for Disease Control and Prevention. Low HDL-C follows the American Heart Association (AHA) cutpoints for metabolic syndrome and TC levels were considered poor based on the definition from the AHA 2021 updated recommendations for cardiovascular health. HbA1c cutpoints follow glycemic goals established by the American Diabetes Association for diagnosis of diabetes. | ≤ 3 mg/L = 0, > 3 mg/L = 1 |
| High-density lipoprotein cholesterol (HDL) |  | Men: ≥ 40 mg/dL = 0, < 40 mg/dL = 1  Women: ≥ 50 mg/dL = 0, < 50 mg/dL = 1 |
| Total cholesterol (TC) |  | < 200 mg/dL = 0, ≥ 200 mg/dL = 1 |
| Glycosylated haemoglobin A1c (HbA1c) |  | < 6.5% = 0, ≥ 6.5% = 1 |
| Apolipoprotein E (APOE) gene | APOE genotype was assessed from saliva samples collected during home visits. A random half of the participants provided samples in 2006; the other half in 2008. Saliva-collection participation rates were 83% in 2006 and 84% in 2008. Genotyping was performed by the National Institute of Health Center for Inherited Disease Research, and then archived by the National Center for Biotechnology Information. | Non-ε4 carriers = 0, Heterozygous ɛ4 carriers = 1, Homozygous ɛ4 carriers = 2 |

Notes: Frailty indicators must meet the following criteria: (1) Variables included in the frailty index must have less than 5% missing data. (2) Each variable must represent a health-related impairment with a prevalence exceeding 1% in the study population. (3) The prevalence of health impairments should generally increase with advancing age. (4) Health deficits should not be prematurely saturated, defined as having a prevalence exceeding 80%. (5) Frailty indicators must cover a broad range of health domains and reflect overall system-wide health status rather than being restricted to specific systems[4].

All self-reported diseases were clinically diagnosed at secondary-level or higher hospitals.

Memory-related disease included Alzheimer’s disease, dementia, organic brain senility, and other serious memory impairment.

The memory score was the average of words which were not recalled in the immediate and delayed word recall tasks. The memory score ranged from 0 to 10. The orientation test comprised 4 questions about the day of the week, the month, the date of the month, and the year. One point was given for each wrong answer, and the range was from 0 to 4.

**Table S2** Motoric cognitive risk frailty syndrome with transitions among non-frailty, frailty, and death in the multi-state Markov model, HR (95%CI) (n = 10809)

| Transitions | Motoric cognitive risk syndrome | | | | | | | |
| --- | --- | --- | --- | --- | --- | --- | --- | --- |
|  | Non-MCR | MCR |  |  | Normal | SCC only | Slow gait speed only | MCR |
| State 1 to state 2 | Ref. | **1.39 (1.20-1.61)** |  |  | Ref. | **1.36 (1.28-1.45)** | 1.10 (0.97-1.25) | **1.56 (1.34-1.81)** |
| State 1 to state 3 | Ref. | 1.04 (0.07-15.63) |  |  | Ref. | 0.53 (0.21-1.32) | 1.36 (0.51-3.60) | 0.73 (0.07-8.17) |
| State 2 to state 1 | Ref. | **0.63 (0.52-0.77)** |  |  | Ref. | **0.76 (0.70-0.84)** | **0.70 (0.58-0.85)** | **0.53 (0.43-0.65)** |
| State 2 to state 3 | Ref. | 0.99 (0.88-1.12) |  |  | Ref. | **0.88 (0.81-0.95)** | 1.09 (0.95-1.24) | 0.95 (0.83-1.09) |

Notes: Multi-state Markov model: using the new cutoff value of frailty index (0.21); HR: hazard ratio after adjusting for age, sex, race, place of residence, educational level, marital status, per capita household income, smoking status, alcohol consumption, physical activity, social participation, body mass index, waist circumference, pain, tooth loss, hospitalization history, medication use, hand grip strength, peak expiratory flow, systolic blood pressure, diastolic blood pressure, high-density lipoprotein cholesterol, total cholesterol, C-reactive protein, glycosylated haemoglobin A1c, apolipoprotein E gene, and baseline assessment time; CI: confidence interval; SCC: subjective cognitive complaints; MCR: motoric cognitive risk syndrome; Boldface: *P* < 0.05.

State 1: non-frailty; State 2: frailty; State 3: death

**Table S3** Associations of motoric cognitive risk syndrome with frailty risk and its reversibility in the Cox proportional hazard regression models, HR (95%CI)

| Motoric cognitive risk syndrome | Frailty (n = 6973) | Frailty reversibility (n = 3836) |
| --- | --- | --- |
| Non-MCR | Ref. | Ref. |
| MCR | **1.31 (1.09-1.58)** | **0.52 (0.40-0.68)** |
|  |  |  |
| Normal | Ref. | Ref. |
| Subjective cognitive complaints only | **1.43 (1.34-1.54)** | **0.73 (0.63-0.84)** |
| Slow gait speed only | **1.16 (1.01-1.35)** | **0.68 (0.52-0.88)** |
| MCR | **1.49 (1.23-1.80)** | **0.43 (0.32-0.56)** |

Notes: Cox regression analysis: using the new cutoff value of frailty index (0.21); HR: hazard ratio; CI: confidence interval; MCR: motoric cognitive risk syndrome; Boldface: *P* < 0.05.

With frailty as the outcome, hazard ratios were adjusted for age, sex, race, educational level, marital status, per capita household income, smoking status, alcohol consumption, physical activity, social participation, body mass index, waist circumference, pain, tooth loss, hospitalization history, medication use, hand grip strength, peak expiratory flow, systolic blood pressure, high-density lipoprotein cholesterol, total cholesterol, C-reactive protein, glycosylated haemoglobin A1c, and baseline assessment time;

With non-frailty as the outcome, hazard ratios were adjusted for age, place of residence, educational level, marital status, per capita household income, alcohol consumption, physical activity, social participation, body mass index, pain, hospitalization history, medication use, hand grip strength, peak expiratory flow, total cholesterol, and glycosylated haemoglobin A1c.

**Table S4** Associations of motoric cognitive risk syndrome with frailty risk and its reversibility in the Cox proportional hazard regression models, HR (95%CI)

| Motoric cognitive risk syndrome | Frailty (n = 6810) | Frailty reversibility (n = 1803) |
| --- | --- | --- |
| Non-MCR | Ref. | Ref. |
| MCR | **1.48 (1.21-1.81)** | **0.46 (0.30-0.71)** |
|  |  |  |
| Normal | Ref. | Ref. |
| Subjective cognitive complaints only | **1.47 (1.35-1.60)** | 0.89 (0.67-1.19) |
| Slow gait speed only | 1.17 (0.98-1.39) | 1.23 (0.82-1.85) |
| MCR | **1.72 (1.40-2.11)** | **0.45 (0.28-0.71)** |

Notes: Cox regression analysis: excluding participants with less than three years of follow-up; HR: hazard ratio; CI: confidence interval; MCR: motoric cognitive risk syndrome; Boldface: *P* < 0.05.

With frailty as the outcome, hazard ratios were adjusted for age, race, place of residence, educational level, marital status, per capita household income, smoking status, alcohol consumption, physical activity, social participation, body mass index, waist circumference, pain, tooth loss, hospitalization history, medication use, hand grip strength, peak expiratory flow, systolic blood pressure, high-density lipoprotein cholesterol, total cholesterol, C-reactive protein, glycosylated haemoglobin A1c, and baseline assessment time;

With non-frailty as the outcome, hazard ratios were adjusted for age, marital status, per capita household income, physical activity, pain, medication use, hand grip strength, peak expiratory flow, and total cholesterol.

**Table S5** Motoric cognitive risk frailty syndrome with transitions among robustness, pre-frailty, frailty, and death in the multi-state Markov model, HR (95%CI) (n = 10809)

| Transitions | Motoric cognitive risk syndrome | | | | | | | |
| --- | --- | --- | --- | --- | --- | --- | --- | --- |
|  | Non-MCR | MCR |  |  | Normal | SCC only | Slow gait speed only | MCR |
| State 1 to state 2 | Ref. | 0.98 (0.75-1.27) |  |  | Ref. | **1.34 (1.23-1.46)** | **1.21 (1.01-1.44)** | 1.07 (0.82-1.39) |
| State 1 to state 4 | Ref. | 0.41 (0.04-4.02) |  |  | Ref. | 1.88 (0.93-3.80) | 1.19 (0.18-7.78) | 0.48 (0.05-4.88) |
| State 2 to state 1 | Ref. | **0.60 (0.41-0.87)** |  |  | Ref. | **0.89 (0.79-0.99)** | 0.90 (0.70-1.16) | **0.57 (0.39-0.82)** |
| State 2 to state 3 | Ref. | **1.57 (1.37-1.78)** |  |  | Ref. | **1.32 (1.24-1.41)** | 1.12 (0.98-1.27) | **1.77 (1.54-2.04)** |
| State 2 to state 4 | Ref. | 0.66 (0.11-3.74) |  |  | Ref. | **0.20 (0.05-0.85)** | 1.20 (0.60-2.41) | 0.30 (0.02-5.18) |
| State 3 to state 2 | Ref. | **0.63 (0.52-0.77)** |  |  | Ref. | **0.81 (0.73-0.90)** | **0.68 (0.56-0.82)** | **0.54 (0.44-0.66)** |
| State 3 to state 4 | Ref. | 0.94 (0.82-1.06) |  |  | Ref. | **0.86 (0.79-0.94)** | 1.03 (0.89-1.19) | 0.88 (0.76-1.01) |

Notes: When we constructed the four-state Markov model to analysis the association between motoric cognitive risk syndrome and transitions among robustness, pre-frailty, frailty, and death, only 214 transitions from robustness to frailty and 15 transitions from frailty to robustness across 8 waves. To ensure the robustness of the model, the direct transitions between robustness and frailty were not allowed in our analysis.

HR: hazard ratio after adjusting for age, sex, race, educational level, marital status, per capita household income, smoking status, alcohol consumption, physical activity, social participation, body mass index, tooth loss, hospitalization history, medication use, hand grip strength, peak expiratory flow, systolic blood pressure, high-density lipoprotein cholesterol, total cholesterol, C-reactive protein, glycosylated haemoglobin A1c, and apolipoprotein E gene; CI: confidence interval; SCC: subjective cognitive complaints; MCR: motoric cognitive risk syndrome; Boldface: *P* < 0.05.

State 1: robustness; State 2: pre-frailty; State 3: frailty; State 4: death

**Table S6** Associations of motoric cognitive risk syndrome with transitions among robustness, pre-frailty, and frailty in the Cox proportional hazard regression models, HR (95%CI)

| Motoric cognitive risk syndrome | Robustness at baseline (n = 2843) | |  | Pre-frailty at baseline (n = 5315) | |  | Frailty at baseline (n = 2651) |
| --- | --- | --- | --- | --- | --- | --- | --- |
|  | Pre-frailty risk ^a^ | Frailty risk ^b^ |  | Robustness likelihood ^c^ | Frailty risk ^d^ |  | Pre-frailty likelihood ^e^ |
| Non-MCR | Ref. | Ref. |  | Ref. | Ref. |  | Ref. |
| MCR | 1.05 (0.75-1.47) | **1.99 (1.26-3.16)** |  | 0.64 (0.40-1.03) | **1.35 (1.13-1.61)** |  | **0.55 (0.43-0.71)** |
|  |  |  |  |  |  |  |  |
| Normal | Ref. | Ref. |  | Ref. | Ref. |  | Ref. |
| Subjective cognitive complaints only | **1.27 (1.14-1.41)** | **1.38 (1.14-1.67)** |  | 0.87 (0.75-1.01) | **1.32 (1.22-1.42)** |  | **0.81 (0.68-0.96)** |
| Slow gait speed only | 1.03 (0.82-1.28) | 1.36 (0.95-1.94) |  | 0.93 (0.66-1.29) | 1.15 (0.99-1.35) |  | **0.75 (0.57-0.99)** |
| MCR | 1.11 (0.79-1.56) | **2.23 (1.40-3.56)** |  | **0.61 (0.38-0.97)** | **1.52 (1.27-1.82)** |  | **0.48 (0.37-0.62)** |

Notes: ^a^: Hazard ratio after adjusting for age, educational level, per capita household income, alcohol consumption, physical activity, body mass index, waist circumference, pain, hospitalization history, medication use, systolic blood pressure, C-reactive protein, glycosylated haemoglobin A1c, and baseline assessment time;

^b^: Hazard ratio after adjusting for age, race, educational level, marital status, per capita household income, smoking status, alcohol consumption, physical activity, social participation, body mass index, waist circumference, pain, tooth loss, hospitalization history, hand grip strength, peak expiratory flow, systolic blood pressure, high-density lipoprotein cholesterol, C-reactive protein, glycosylated haemoglobin A1c, apolipoprotein E gene, and baseline assessment time;

^c^ Hazard ratio after adjusting for age, marital status, per capita household income, alcohol consumption, physical activity, body mass index, waist circumference, pain, hospitalization history, medication use, hand grip strength, peak expiratory flow, high-density lipoprotein cholesterol, glycosylated haemoglobin A1c, and baseline assessment time;

^d^: Hazard ratio after adjusting for age, educational level, marital status, per capita household income, smoking status, alcohol consumption, physical activity, social participation, body mass index, waist circumference, pain, tooth loss, hospitalization history, medication use, hand grip strength, peak expiratory flow, systolic blood pressure, diastolic blood pressure, high-density lipoprotein cholesterol, C-reactive protein, glycosylated haemoglobin A1c, and baseline assessment time;

^e^: Hazard ratio after adjusting for age, sex, place of residence, educational level, marital status, per capita household income, alcohol consumption, physical activity, social participation, body mass index, pain, tooth loss, hospitalization history, medication use, hand grip strength, peak expiratory flow, total cholesterol, and glycosylated haemoglobin A1c;

When we used robustness as the outcome to construct cohorts with frail participants at baseline (n = 2651), only 11 participants reversed to robustness. Thus, the Cox proportional hazard regression model was not conducted.

HR: hazard ratio; CI: confidence interval; MCR: motoric cognitive risk syndrome; Boldface: *P* < 0.05.

**Table S7** Motoric cognitive risk frailty syndrome with transitions among non-frailty, frailty, and death in the multi-state Markov model, HR (95%CI) (n = 9160)

| Transitions | Motoric cognitive risk syndrome | | | | | | | |
| --- | --- | --- | --- | --- | --- | --- | --- | --- |
|  | Non-MCR | MCR |  |  | Normal | SCC only | Slow gait speed only | MCR |
| State 1 to state 2 | Ref. | **1.49 (1.27-1.75)** |  |  | Ref. | **1.50 (1.40-1.62)** | **1.21 (1.05-1.40)** | **1.76 (1.49-2.08)** |
| State 1 to state 3 | Ref. | 1.46 (0.56-3.82) |  |  | Ref. | **0.54 (0.30-0.97)** | 0.81 (0.33-1.98) | 1.15 (0.43-3.09) |
| State 2 to state 1 | Ref. | **0.71 (0.57-0.89)** |  |  | Ref. | **0.85 (0.76-0.95)** | **0.69 (0.55-0.87)** | **0.63 (0.50-0.79)** |
| State 2 to state 3 | Ref. | 0.96 (0.81-1.13) |  |  | Ref. | **0.88 (0.80-0.98)** | 1.04 (0.87-1.23) | 0.91 (0.77-1.09) |

Notes: Multi-state Markov model: excluding participants with mild cognitive impairment at baseline; HR: hazard ratio after adjusting for age, sex, race, place of residence, educational level, marital status, per capita household income, smoking status, alcohol consumption, physical activity, social participation, body mass index, waist circumference, pain, tooth loss, hospitalization history, medication use, hand grip strength, peak expiratory flow, systolic blood pressure, diastolic blood pressure, high-density lipoprotein cholesterol, total cholesterol, C-reactive protein, glycosylated haemoglobin A1c, apolipoprotein E gene, and baseline assessment time; CI: confidence interval; SCC: subjective cognitive complaints; MCR: motoric cognitive risk syndrome; Boldface: *P* < 0.05.

State 1: non-frailty; State 2: frailty; State 3: death

**Table S8** Associations of motoric cognitive risk syndrome with frailty risk and its reversibility in the Cox proportional hazard regression models, HR (95%CI)

| Motoric cognitive risk syndrome | Frailty (n = 7250) | Frailty reversibility (n = 1910) |
| --- | --- | --- |
| Non-MCR | Ref. | Ref. |
| MCR | **1.37 (1.13-1.67)** | **0.57 (0.42-0.77)** |
|  |  |  |
| Normal | Ref. | Ref. |
| Subjective cognitive complaints only | **1.52 (1.41-1.65)** | **0.80 (0.66-0.97)** |
| Slow gait speed only | **1.30 (1.11-1.52)** | 0.74 (0.53-1.02) |
| MCR | **1.64 (1.34-2.00)** | **0.49 (0.35-0.67)** |

Notes: Cox regression analysis: excluding participants with mild cognitive impairment at baseline; HR: hazard ratio; CI: confidence interval; MCR: motoric cognitive risk syndrome;; Boldface: *P* < 0.05.

With frailty as the outcome, hazard ratios were adjusted for age, sex, race, educational level, marital status, per capita household income, smoking status, alcohol consumption, physical activity, social participation, body mass index, waist circumference, pain, tooth loss, hospitalization history, medication use, hand grip strength, peak expiratory flow, systolic blood pressure, high-density lipoprotein cholesterol, total cholesterol, C-reactive protein, glycosylated haemoglobin A1c, and baseline assessment time;

With non-frailty as the outcome, hazard ratios were adjusted for age, educational level, marital status, per capita household income, alcohol consumption, physical activity, body mass index, pain, hospitalization history, medication use, hand grip strength, peak expiratory flow, total cholesterol, and glycosylated haemoglobin A1c.

**Table S9** Motoric cognitive risk frailty syndrome with transitions among non-frailty, frailty, and death in the multi-state Markov model, HR (95%CI) (n = 10809)

| Transitions | Motoric cognitive risk syndrome | | | | | | | |
| --- | --- | --- | --- | --- | --- | --- | --- | --- |
|  | Non-MCR | MCR |  |  | Normal | SCC only | Slow gait speed only | MCR |
| State 1 to state 2 | Ref. | **1.47 (1.36-1.58)** |  |  | Ref. | **1.48 (1.36-1.62)** | **1.31 (1.21-1.43)** | **1.80 (1.64-1.98)** |
| State 1 to state 3 | Ref. | 0.75 (0.36-1.58) |  |  | Ref. | 0.74 (0.36-1.52) | 1.09 (0.70-1.71) | 0.63 (0.23-1.75) |
| State 2 to state 1 | Ref. | **0.82 (0.74-0.92)** |  |  | Ref. | **0.77 (0.67-0.88)** | **0.80 (0.69-0.92)** | **0.68 (0.59-0.79)** |
| State 2 to state 3 | Ref. | **0.91 (0.84-0.99)** |  |  | Ref. | **0.86 (0.74-0.98)** | 1.05 (0.93-1.18) | 0.90 (0.80-1.03) |

Notes: Multi-state Markov model: using 0.80 m/s as the cutoff value of slow gait speed; HR: hazard ratio after adjusting for age, sex, race, place of residence, educational level, marital status, per capita household income, smoking status, alcohol consumption, physical activity, social participation, body mass index, waist circumference, pain, tooth loss, hospitalization history, medication use, hand grip strength, peak expiratory flow, systolic blood pressure, diastolic blood pressure, high-density lipoprotein cholesterol, total cholesterol, C-reactive protein, glycosylated haemoglobin A1c, apolipoprotein E gene, and baseline assessment time; CI: confidence interval; SCC: subjective cognitive complaints; MCR: motoric cognitive risk syndrome; Boldface: *P* < 0.05.

State 1: non-frailty; State 2: frailty; State 3: death

**Table S10** Associations of motoric cognitive risk syndrome with frailty risk and its reversibility in the Cox proportional hazard regression models, HR (95%CI)

| Motoric cognitive risk syndrome | Frailty (n = 8158) | Frailty reversibility (n = 2651) |
| --- | --- | --- |
| Non-MCR | Ref. | Ref. |
| MCR | **1.43 (1.31-1.56)** | **0.76 (0.65-0.89)** |
|  |  |  |
| Normal | Ref. | Ref. |
| Subjective cognitive complaints only | **1.58 (1.44-1.73)** | **0.73 (0.58-0.92)** |
| Slow gait speed only | **1.38 (1.26-1.51)** | 0.81 (0.64-1.02) |
| MCR | **1.80 (1.63-1.99)** | **0.62 (0.49-0.78)** |

Notes: Cox regression analysis: using 0.80 m/s as the cutoff value of slow gait speed; HR: hazard ratio; CI: confidence interval; MCR: motoric cognitive risk syndrome;; Boldface: *P* < 0.05.

With frailty as the outcome, hazard ratios were adjusted for age, sex, race, place of residence, educational level, marital status, per capita household income, smoking status, alcohol consumption, physical activity, social participation, body mass index, waist circumference, pain, tooth loss, hospitalization history, medication use, hand grip strength, peak expiratory flow, systolic blood pressure, high-density lipoprotein cholesterol, total cholesterol, C-reactive protein, glycosylated haemoglobin A1c, and baseline assessment time;

With non-frailty as the outcome, hazard ratios were adjusted for age, sex, race, place of residence, educational level, marital status, per capita household income, alcohol consumption, physical activity, social participation, body mass index, pain, tooth loss, hospitalization history, medication use, hand grip strength, peak expiratory flow, total cholesterol, and glycosylated haemoglobin A1c.

**References:**

1.Martinez M, Dawson A Z, Lu K, Walker R J, Egede L E. Effect of cognitive impairment on risk of death in hispanic/latino adults over the age of 50 residing in the united states with and without diabetes: data from the health and retirement study 1995-2014. Alzheimers Dement. 2022;18:1616-1624. doi:10.1002/alz.12521

2.Wu C K, Geldhof G J, Xue Q L, Kim D H, Newman A B, Odden M C. Development, construct validity, and predictive validity of a continuous frailty scale: results from 2 large us cohorts. Am J Epidemiol. 2018;187:1752-1762. doi:10.1093/aje/kwy041

3.Yu K X, Wu S Y, Chi I. Internet use and loneliness of older adults over time: the mediating effect of social contact. J Gerontol B-Psychol. 2021;76:541-550. doi:10.1093/geronb/gbaa004

4.Theou O, Haviva C, Wallace L, Searle S D, Rockwood K. How to construct a frailty index from an existing dataset in 10 steps. Age Ageing. 2023;52. doi:10.1093/ageing/afad221
