# Supplementary material for: Motoric Cognitive Risk Syndrome Associated With Risk of Frailty and Likelihood of Reversion in Older Adults
Source: J Cachexia Sarcopenia Muscle. 2025 Jul 29;16(4):e70033. doi: 10.1002/jcsm.70033 (PMC12304730; doi:10.1002/jcsm.70033)
Supplement: Supplementary file 2 — Figure S1 Selection flow of the study population in the HRS. [file JCSM-16-e70033-s002.docx]

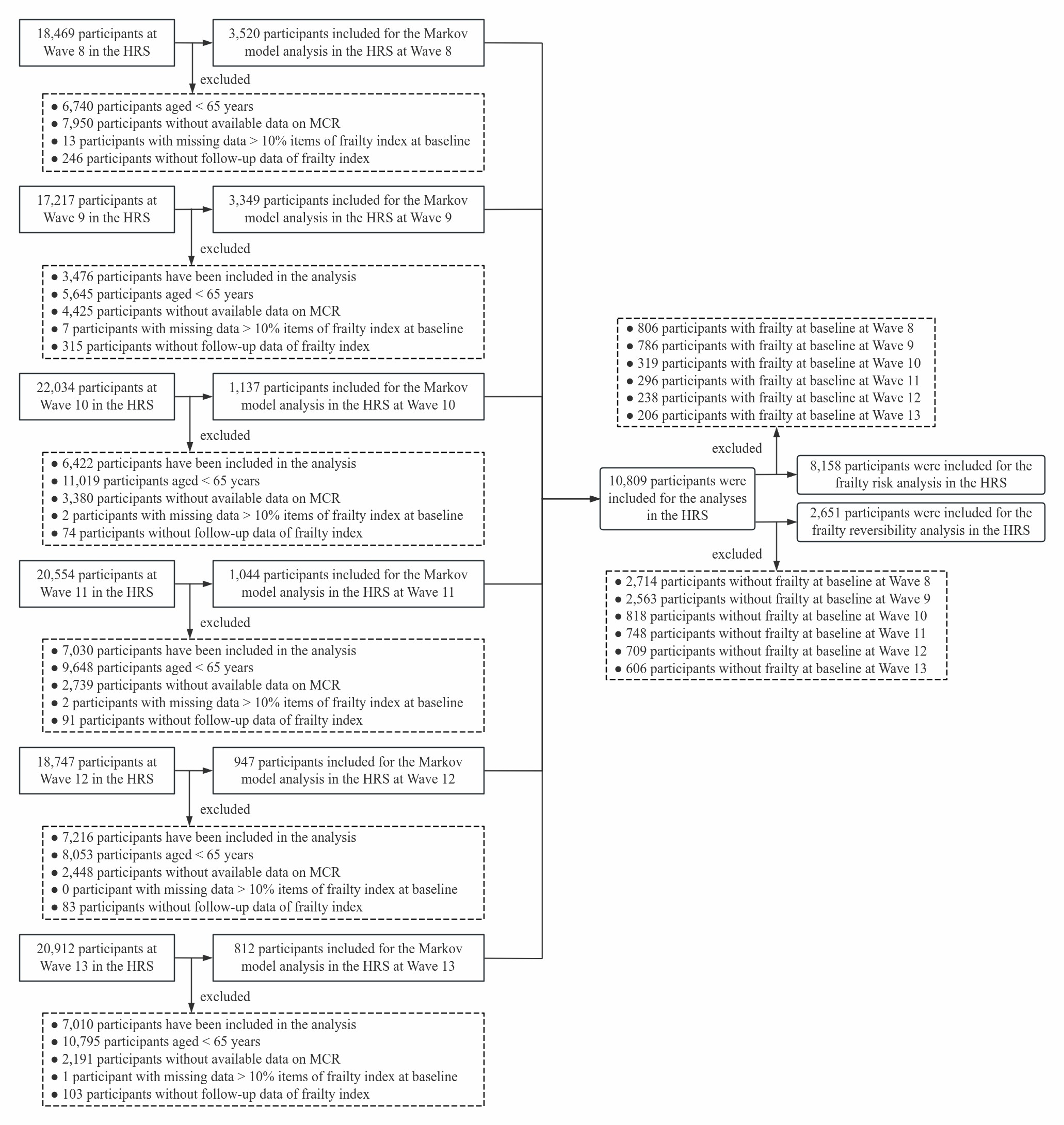


**Figure S1** Selection flow of the study population in the HRS.

Notes: HRS: Health and Retirement Study; MCR: motoric cognitive risk syndrome
